# Supplementary figures and images for: Resveratrol’s Impact on the Chondrogenic Reagents’ Effects in Cell Sheet Cultures of Wharton’s Jelly-Derived MSCs
Source: Cells. 2023 Dec 15;12(24):2845. doi: 10.3390/cells12242845 (PMC10741663; doi:10.3390/cells12242845)

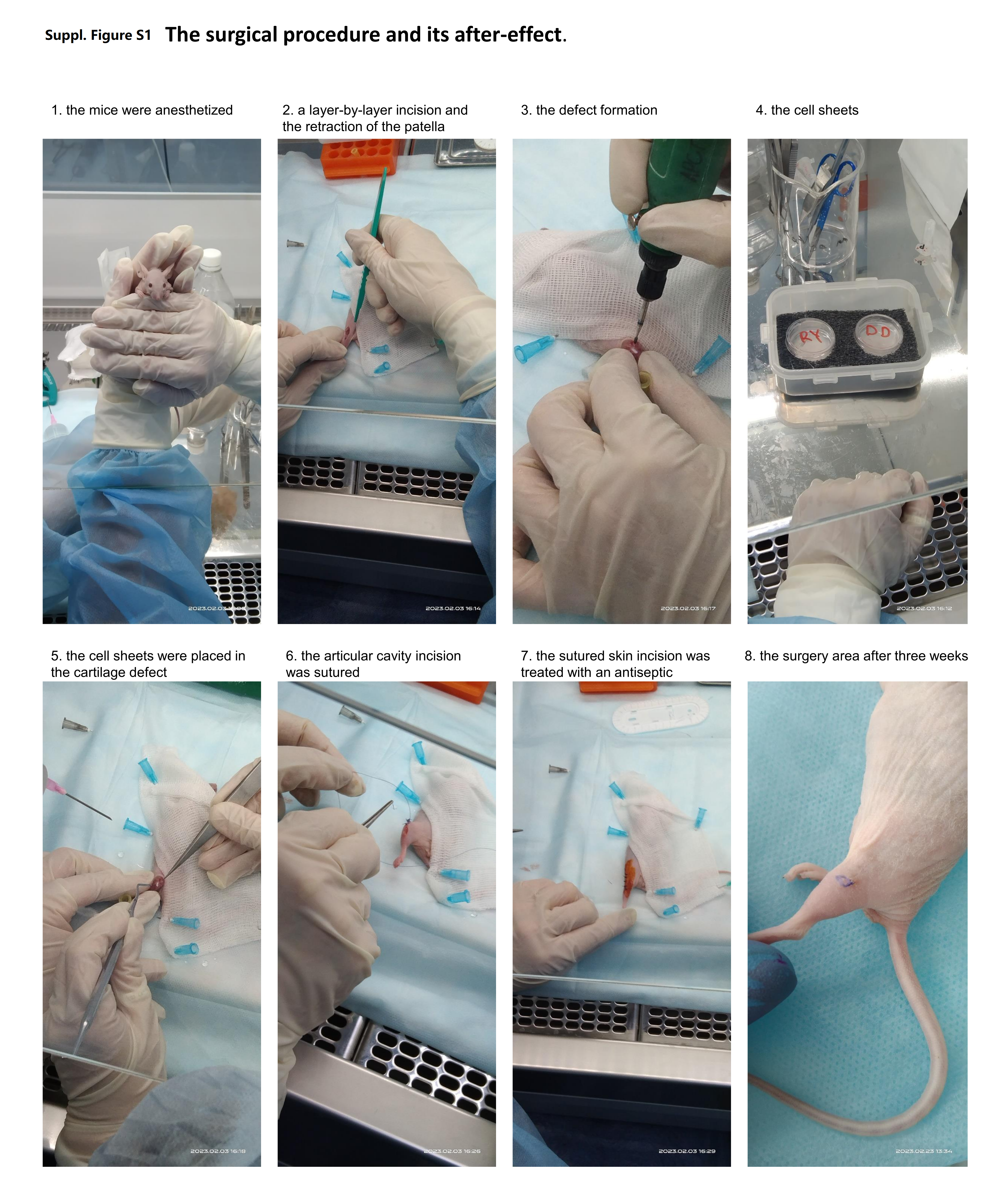

Supplement: Supplementary file 1 [file cells-12-02845-s001.zip › Supplementary Figure S1.tif]
